# (*E*)-1-(4-Nitro­phen­yl)-3-phenyl­prop-2-en-1-one

**DOI:** 10.1107/S1600536809037556

**Published:** 2009-09-26

**Authors:** Lin-Hai Jing

**Affiliations:** a School of Chemistry and Chemical Engineering, China West Normal University, Nanchong 637002, People’s Republic of China

## Abstract

In the title compound, C_15_H_11_NO_3_, the configuration of the keto group with respect to the olefinic double bond is *s–cis*. The two benzene rings form a dihedral angle of 5.00 (5)°. The mol­ecules are linked into a two-dimensional network parallel to (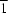
04) by C—H⋯O hydrogen bonds.

## Related literature

For the biological activity of chalcone derivatives, see: Dimmock *et al.* (1999 ▶). For the synthesis, see: Cocconcelli *et al.* (2008 ▶).
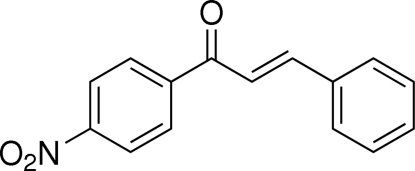


## Experimental

### 

#### Crystal data

C_15_H_11_NO_3_
*M* *_r_* = 253.25
Monoclinic, 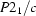

*a* = 6.2139 (10) Å
*b* = 13.159 (2) Å
*c* = 14.450 (3) Å
β = 92.106 (3)°
*V* = 1180.8 (3) Å^3^
*Z* = 4
Mo *K*α radiation
μ = 0.10 mm^−1^
*T* = 93 K
0.50 × 0.18 × 0.18 mm

#### Data collection

Rigaku SPIDER diffractometer
Absorption correction: none
9403 measured reflections
2687 independent reflections
2264 reflections with *I* > 2σ(*I*)
*R*_int_ = 0.025

#### Refinement

*R*[*F* ^2^ > 2σ(*F* ^2^)] = 0.037
*wR*(*F* ^2^) = 0.097
*S* = 1.00
2687 reflections
172 parameters
H-atom parameters constrained
Δρ_max_ = 0.36 e Å^−3^
Δρ_min_ = −0.19 e Å^−3^

### 

Data collection: *RAPID-AUTO* (Rigaku, 2004 ▶); cell refinement: *RAPID-AUTO*; data reduction: *RAPID-AUTO*; program(s) used to solve structure: *SHELXS97* (Sheldrick, 2008 ▶); program(s) used to refine structure: *SHELXL97* (Sheldrick, 2008 ▶); molecular graphics: *XP* in *SHELXTL* (Sheldrick, 2008 ▶); software used to prepare material for publication: *SHELXL97*.

## Supplementary Material

Crystal structure: contains datablocks global, I. DOI: 10.1107/S1600536809037556/ci2913sup1.cif

Structure factors: contains datablocks I. DOI: 10.1107/S1600536809037556/ci2913Isup2.hkl

Additional supplementary materials:  crystallographic information; 3D view; checkCIF report

## Figures and Tables

**Table 1:** Hydrogen-bond geometry (Å, °)

*D*—H⋯*A*	*D*—H	H⋯*A*	*D*⋯*A*	*D*—H⋯*A*
C1—H1⋯O1^i^	0.95	2.47	3.3314 (15)	150
C5—H5⋯O3^ii^	0.95	2.56	3.4635 (16)	160

Symmetry codes: (i) 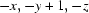; (ii) 
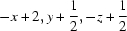
.

**Fig. 1.**
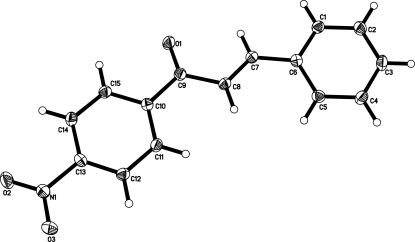
The molecular structure of the title compound, showing 50% probability displacement ellipsoids and the atomic numbering.

## supplementary crystallographic information

### Comment

Chalcone derivatives are a class of important compounds that possess antiprotzoal, antihelmintic, amoebicidal, anti-ulcer, antiviral, insecticidal, antibacterial, anticancer, cytotoxic and immunosuppressive activities (Dimmock *et al.*, 1999). The crystal structures of some chalcone derivatives have been reported. We report here the crystal structure of the title compound.

Bond lengths and angles in the title molecule are normal. The configuration of the keto group with respect to the olefinic double bond is typically *s-cis*, with a O1—C9—C8—C7 torsion angle of 0.5 (2)° (Fig. 1). The N1/O2/O3 and C10-C15 planes form a dihedral angle of 10.80 (11)°. The two benzene rings (C10-C15 and C1-C6) form a dihedral angle of 5.00 (5)°. The crystal packing is stabilized by C—H···O hydrogen bonds (Table 1). The molecules are linked into a two-dimensional network parallel to the (1 0 4) by the above hydrogen bonds.

### Experimental

The title compound was synthesized according to the method reported in the literature (Cocconcelli *et al.*, 2008). Yellow single crystals suitable for X-ray diffraction were obtained by slow evaporation of a acetone solution.

### Refinement

All H atoms were placed in calculated positions, with C-H = 0.95 Å, and refined using a riding model, with *U*_iso_(H) = 1.2*U*_eq_(C).

### Crystal data

**Table:**

C_15_H_11_NO_3_	*F*(000) = 528
*M**_r_* = 253.25	*D*_x_ = 1.425 Mg m^−^^3^
Monoclinic, *P*2_1_/*c*	Mo *K*α radiation, λ = 0.71073 Å
Hall symbol: -P 2ybc	Cell parameters from 3448 reflections
*a* = 6.2139 (10) Å	θ = 3.1–27.5°
*b* = 13.159 (2) Å	µ = 0.10 mm^−^^1^
*c* = 14.450 (3) Å	*T* = 93 K
β = 92.106 (3)°	Needle, yellow
*V* = 1180.8 (3) Å^3^	0.50 × 0.18 × 0.18 mm
*Z* = 4	

### Data collection

**Table:**

Rigaku SPIDER diffractometer	2264 reflections with *I* > 2σ(*I*)
Radiation source: fine-focus sealed tube	*R*_int_ = 0.025
graphite	θ_max_ = 27.5°, θ_min_ = 3.1°
ω scans	*h* = −8→7
9403 measured reflections	*k* = −17→17
2687 independent reflections	*l* = −18→15

### Refinement

**Table:**

Refinement on *F*^2^	Primary atom site location: structure-invariant direct methods
Least-squares matrix: full	Secondary atom site location: difference Fourier map
*R*[*F*^2^ > 2σ(*F*^2^)] = 0.037	Hydrogen site location: inferred from neighbouring sites
*wR*(*F*^2^) = 0.097	H-atom parameters constrained
*S* = 1.00	*w* = 1/[σ^2^(*F*_o_^2^) + (0.0516*P*)^2^ + 0.336*P*] where *P* = (*F*_o_^2^ + 2*F*_c_^2^)/3
2687 reflections	(Δ/σ)_max_ = 0.001
172 parameters	Δρ_max_ = 0.36 e Å^−^^3^
0 restraints	Δρ_min_ = −0.19 e Å^−^^3^

### Special details

**Table:**

Geometry. All e.s.d.'s (except the e.s.d. in the dihedral angle between two l.s. planes) are estimated using the full covariance matrix. The cell e.s.d.'s are taken into account individually in the estimation of e.s.d.'s in distances, angles and torsion angles; correlations between e.s.d.'s in cell parameters are only used when they are defined by crystal symmetry. An approximate (isotropic) treatment of cell e.s.d.'s is used for estimating e.s.d.'s involving l.s. planes.
Refinement. Refinement of *F*^2^ against ALL reflections. The weighted *R*-factor *wR* and goodness of fit *S* are based on *F*^2^, conventional *R*-factors *R* are based on *F*, with *F* set to zero for negative *F*^2^. The threshold expression of *F*^2^ > σ(*F*^2^) is used only for calculating *R*-factors(gt) *etc*. and is not relevant to the choice of reflections for refinement. *R*-factors based on *F*^2^ are statistically about twice as large as those based on *F*, and *R*- factors based on ALL data will be even larger.

### Fractional atomic coordinates and isotropic or equivalent isotropic displacement parameters (Å^2^)

**Table:**

	*x*	*y*	*z*	*U*_iso_*/*U*_eq_	
O1	0.19885 (14)	0.39882 (7)	0.06264 (7)	0.0243 (2)	
O2	0.89968 (15)	0.02961 (7)	0.20944 (7)	0.0266 (2)	
O3	1.11753 (14)	0.14090 (7)	0.27163 (7)	0.0272 (2)	
N1	0.94974 (16)	0.11770 (8)	0.22851 (7)	0.0185 (2)	
C1	0.11212 (19)	0.77346 (9)	0.04561 (8)	0.0179 (3)	
H1	−0.0164	0.7395	0.0258	0.021*	
C2	0.1184 (2)	0.87899 (9)	0.04625 (9)	0.0209 (3)	
H2	−0.0059	0.9169	0.0277	0.025*	
C3	0.3061 (2)	0.92892 (9)	0.07402 (9)	0.0221 (3)	
H3	0.3104	1.0011	0.0741	0.027*	
C4	0.4885 (2)	0.87379 (9)	0.10183 (8)	0.0205 (3)	
H4	0.6170	0.9083	0.1206	0.025*	
C5	0.48224 (19)	0.76832 (9)	0.10203 (8)	0.0180 (3)	
H5	0.6066	0.7309	0.1214	0.022*	
C6	0.29377 (19)	0.71675 (9)	0.07389 (8)	0.0158 (3)	
C7	0.27514 (19)	0.60597 (9)	0.07315 (8)	0.0163 (2)	
H7	0.1469	0.5789	0.0451	0.020*	
C8	0.41785 (19)	0.53830 (9)	0.10745 (8)	0.0169 (3)	
H8	0.5496	0.5604	0.1362	0.020*	
C9	0.36764 (19)	0.42863 (9)	0.10009 (8)	0.0167 (3)	
C10	0.52508 (19)	0.35096 (9)	0.13838 (8)	0.0155 (2)	
C11	0.72971 (19)	0.37564 (9)	0.17453 (8)	0.0173 (3)	
H11	0.7733	0.4448	0.1780	0.021*	
C12	0.87037 (19)	0.29959 (9)	0.20556 (8)	0.0172 (3)	
H12	1.0095	0.3158	0.2308	0.021*	
C13	0.80212 (19)	0.19972 (9)	0.19868 (8)	0.0158 (3)	
C14	0.59903 (19)	0.17265 (9)	0.16446 (8)	0.0174 (3)	
H14	0.5558	0.1034	0.1618	0.021*	
C15	0.46102 (19)	0.24900 (9)	0.13438 (8)	0.0172 (3)	
H15	0.3209	0.2322	0.1107	0.021*	

### Atomic displacement parameters (Å^2^)

**Table:**

	*U*^11^	*U*^22^	*U*^33^	*U*^12^	*U*^13^	*U*^23^
O1	0.0198 (5)	0.0168 (4)	0.0355 (5)	−0.0023 (4)	−0.0093 (4)	0.0018 (4)
O2	0.0289 (5)	0.0153 (4)	0.0349 (5)	0.0044 (4)	−0.0061 (4)	−0.0033 (4)
O3	0.0188 (5)	0.0252 (5)	0.0367 (6)	0.0010 (4)	−0.0095 (4)	0.0031 (4)
N1	0.0180 (5)	0.0183 (5)	0.0190 (5)	0.0018 (4)	0.0005 (4)	0.0008 (4)
C1	0.0174 (6)	0.0176 (6)	0.0184 (6)	−0.0015 (5)	−0.0016 (5)	0.0006 (5)
C2	0.0212 (6)	0.0174 (6)	0.0238 (6)	0.0029 (5)	−0.0032 (5)	0.0021 (5)
C3	0.0278 (7)	0.0132 (5)	0.0250 (6)	−0.0008 (5)	−0.0022 (5)	−0.0002 (5)
C4	0.0206 (6)	0.0186 (6)	0.0221 (6)	−0.0039 (5)	−0.0017 (5)	−0.0014 (5)
C5	0.0168 (6)	0.0190 (6)	0.0181 (6)	0.0007 (5)	−0.0004 (5)	0.0013 (5)
C6	0.0180 (6)	0.0158 (6)	0.0136 (5)	0.0005 (4)	0.0013 (4)	0.0001 (4)
C7	0.0164 (6)	0.0166 (6)	0.0160 (6)	−0.0012 (4)	0.0004 (4)	−0.0004 (4)
C8	0.0157 (6)	0.0161 (6)	0.0187 (6)	−0.0021 (4)	−0.0016 (5)	−0.0009 (4)
C9	0.0169 (6)	0.0161 (6)	0.0170 (6)	−0.0007 (5)	0.0003 (5)	0.0006 (4)
C10	0.0168 (6)	0.0148 (5)	0.0147 (6)	0.0006 (4)	0.0002 (4)	−0.0001 (4)
C11	0.0186 (6)	0.0142 (5)	0.0189 (6)	−0.0019 (5)	−0.0004 (5)	−0.0004 (4)
C12	0.0151 (6)	0.0182 (6)	0.0181 (6)	−0.0015 (4)	−0.0015 (5)	−0.0002 (4)
C13	0.0172 (6)	0.0160 (6)	0.0142 (5)	0.0033 (4)	0.0004 (4)	0.0006 (4)
C14	0.0211 (6)	0.0136 (5)	0.0176 (6)	−0.0007 (5)	−0.0010 (5)	−0.0008 (4)
C15	0.0160 (6)	0.0170 (6)	0.0183 (6)	−0.0018 (4)	−0.0024 (5)	0.0001 (4)

### Geometric parameters (Å, °)

**Table:**

O1—C9	1.2268 (15)	C7—C8	1.3389 (17)
O2—N1	1.2289 (14)	C7—H7	0.95
O3—N1	1.2331 (14)	C8—C9	1.4795 (16)
N1—C13	1.4708 (15)	C8—H8	0.95
C1—C2	1.3893 (17)	C9—C10	1.5060 (16)
C1—C6	1.4014 (17)	C10—C11	1.3951 (17)
C1—H1	0.95	C10—C15	1.4000 (16)
C2—C3	1.3851 (18)	C11—C12	1.3920 (17)
C2—H2	0.95	C11—H11	0.95
C3—C4	1.3923 (18)	C12—C13	1.3835 (17)
C3—H3	0.95	C12—H12	0.95
C4—C5	1.3885 (17)	C13—C14	1.3850 (17)
C4—H4	0.95	C14—C15	1.3808 (16)
C5—C6	1.4009 (17)	C14—H14	0.95
C5—H5	0.95	C15—H15	0.95
C6—C7	1.4624 (16)		
			
O2—N1—O3	123.31 (10)	C7—C8—C9	119.15 (11)
O2—N1—C13	118.46 (10)	C7—C8—H8	120.4
O3—N1—C13	118.23 (10)	C9—C8—H8	120.4
C2—C1—C6	120.54 (11)	O1—C9—C8	121.21 (11)
C2—C1—H1	119.7	O1—C9—C10	118.58 (11)
C6—C1—H1	119.7	C8—C9—C10	120.21 (10)
C3—C2—C1	119.95 (11)	C11—C10—C15	119.50 (11)
C3—C2—H2	120.0	C11—C10—C9	123.39 (11)
C1—C2—H2	120.0	C15—C10—C9	117.09 (11)
C2—C3—C4	120.28 (11)	C12—C11—C10	120.40 (11)
C2—C3—H3	119.9	C12—C11—H11	119.8
C4—C3—H3	119.9	C10—C11—H11	119.8
C5—C4—C3	119.93 (11)	C13—C12—C11	118.21 (11)
C5—C4—H4	120.0	C13—C12—H12	120.9
C3—C4—H4	120.0	C11—C12—H12	120.9
C4—C5—C6	120.44 (11)	C12—C13—C14	122.88 (11)
C4—C5—H5	119.8	C12—C13—N1	119.33 (11)
C6—C5—H5	119.8	C14—C13—N1	117.79 (10)
C5—C6—C1	118.85 (11)	C15—C14—C13	118.19 (11)
C5—C6—C7	123.37 (11)	C15—C14—H14	120.9
C1—C6—C7	117.77 (11)	C13—C14—H14	120.9
C8—C7—C6	127.53 (11)	C14—C15—C10	120.80 (11)
C8—C7—H7	116.2	C14—C15—H15	119.6
C6—C7—H7	116.2	C10—C15—H15	119.6
			
C6—C1—C2—C3	0.74 (19)	C8—C9—C10—C15	175.96 (10)
C1—C2—C3—C4	−0.36 (19)	C15—C10—C11—C12	0.67 (18)
C2—C3—C4—C5	−0.20 (19)	C9—C10—C11—C12	−177.52 (11)
C3—C4—C5—C6	0.38 (18)	C10—C11—C12—C13	0.57 (18)
C4—C5—C6—C1	0.00 (18)	C11—C12—C13—C14	−1.58 (18)
C4—C5—C6—C7	−179.43 (11)	C11—C12—C13—N1	178.29 (10)
C2—C1—C6—C5	−0.56 (18)	O2—N1—C13—C12	−169.34 (11)
C2—C1—C6—C7	178.91 (11)	O3—N1—C13—C12	10.81 (16)
C5—C6—C7—C8	8.14 (19)	O2—N1—C13—C14	10.54 (16)
C1—C6—C7—C8	−171.29 (12)	O3—N1—C13—C14	−169.31 (11)
C6—C7—C8—C9	179.69 (11)	C12—C13—C14—C15	1.28 (18)
C7—C8—C9—O1	0.54 (18)	N1—C13—C14—C15	−178.59 (10)
C7—C8—C9—C10	−179.81 (11)	C13—C14—C15—C10	0.04 (17)
O1—C9—C10—C11	173.86 (12)	C11—C10—C15—C14	−0.99 (18)
C8—C9—C10—C11	−5.80 (17)	C9—C10—C15—C14	177.32 (11)
O1—C9—C10—C15	−4.38 (17)		

### Hydrogen-bond geometry (Å, °)

**Table:**

*D*—H···*A*	*D*—H	H···*A*	*D*···*A*	*D*—H···*A*
C1—H1···O1^i^	0.95	2.47	3.3314 (15)	150
C5—H5···O3^ii^	0.95	2.56	3.4635 (16)	160

Symmetry codes: (i) −*x*, −*y*+1, −*z*; (ii) −*x*+2, *y*+1/2, −*z*+1/2.
